# Supplementary figures and images for: FRET kinase sensor development reveals SnRK2/OST1 activation by ABA but not by MeJA and high CO2 during stomatal closure
Source: eLife. 2020 May 28;9:e56351. doi: 10.7554/eLife.56351 (PMC7289597; doi:10.7554/eLife.56351)

Cropped regions are shown by boxes.


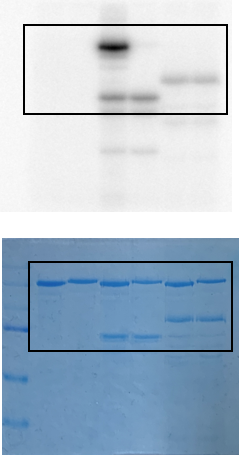

Supplement: Figure 1—source data 1. [file elife-56351-fig1-data1.docx]

Cropped regions are shown by boxes.


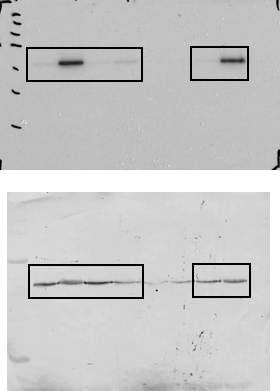

Supplement: Figure 6—figure supplement 1—source data 1. [file elife-56351-fig6-figsupp1-data1.docx]
