# Supplementary material for: FRET kinase sensor development reveals SnRK2/OST1 activation by ABA but not by MeJA and high CO2 during stomatal closure
Source: eLife. 2020 May 28;9:e56351. doi: 10.7554/eLife.56351 (PMC7289597; doi:10.7554/eLife.56351)
Supplement: Supplementary file 1. — Detailed information on the transgenic lines is provided including the plasmid, promoter, and genetic background. [file elife-56351-supp1.docx]

| **Supplementary File 1. Transgenic lines used in the study.** | | | | |
| --- | --- | --- | --- | --- |
| Line name | Plasmid | Promoter | Selection | Background |
| *SNACS/Col-0_1* | 35S: SNACS | CaMV 35S | Hygromycin | Col-0 |
| *SNACS/Col-0_2* | 35S: SNACS | CaMV 35S | Hygromycin | Col-0 |
| *SNACS^S785A^/Col-0_1* | 35S: SNACS^S785A^ | CaMV 35S | Hygromycin | Col-0 |
| *SNACS^S785A^/Col-0_2* | 35S: SNACS^S785A^ | CaMV 35S | Hygromycin | Col-0 |
| *SNACS/OST1-HF_1* | 35S: SNACS | CaMV 35S | Hygromycin | *pUBQ10:OST1-HF/ost1-3* |
| *SNACS/OST1-HF_2* | 35S: SNACS | CaMV 35S | Hygromycin | *pUBQ10:OST1-HF/ost1-3* |
| *SNACS^S785A^/OST1-HF_1* | 35S: SNACS^S785A^ | CaMV 35S | Hygromycin | *pUBQ10:OST1-HF/ost1-3* |
| *SNACS^S785A^/OST1-HF_2* | 35S: SNACS^S785A^ | CaMV 35S | Hygromycin | *pUBQ10:OST1-HF/ost1-3* |
| *SNACS/ost1-3* | 35S: SNACS | CaMV 35S | Hygromycin | *ost1-3* |
| *SNACS/snrk2.2/2.3* | 35S: SNACS | CaMV 35S | Hygromycin | *snrk2.2/2.3* |
| *SNACS/snrk2.2/2.3/2.6* | 35S: SNACS | CaMV 35S | Hygromycin | *snrk2.2/2.3/2.6* |
| *pyl-11458/PYL1* | pGC1: PYL1 | pGC1 | Seed GFP | *pyr1/pyl1458* |
| *pyl-11458/PYL4* | pGC1: PYL4 | pGC1 | Seed GFP | *pyr1/pyl1458* |
| *pyl-11458/PYL5* | pGC1: PYL5 | pGC1 | Seed GFP | *pyr1/pyl1458* |
