# Supplementary material for: FRET kinase sensor development reveals SnRK2/OST1 activation by ABA but not by MeJA and high CO2 during stomatal closure
Source: eLife. 2020 May 28;9:e56351. doi: 10.7554/eLife.56351 (PMC7289597; doi:10.7554/eLife.56351)
Supplement: Supplementary file 2. — Primers used to genotype higher order ABA receptor mutants (Figure 9—figure supplement 3). [file elife-56351-supp2.docx]

| **Supplementary File 2.** **Primer sequences for genotyping.** | | |
| --- | --- | --- |
| Primer | Sequence |  |
| PYR1-F | ACCATGGCTTCGGAGTTAACACCA |  |
| PYR1-R | TCACGTCAC CTGAGAACCACT |  |
| PYL1-F | ATGGCGAATTCAGAGTCCTCC |  |
| PYL1-R | TTACCTAACCTGAGAAGAGTT |  |
| PYL2-F | ACCATGGGCTCATCCCCGGCCGTGA |  |
| PYL2-R | TTATTCATCATCATGCATAGGTG |  |
| PYL4-F | ACCATGGTTGCCGTTCACCGTCCTT |  |
| PYL4-R | TCACAGAGACATCTTCTTCTTGC |  |
| PYL5-LP | AAACACAAAGCCTTCACATCC |  |
| PYL5-RP | AAGTTTTGTGAATCCCCCAAC |  |
| PYL5-R | TTATTGCCGGTTGGTACTTCGA |  |
| PYL8-F | ATGGAAGCTAACGGGATTGAG |  |
| PYL8-R | TTAGACTCTCGATTCTGTCGT |  |
| LBb1.3 | ATTTTGCCGATTTCGGAAC |  |
| Ds5O | GTTCGAATTCGATCGGGATAAAAC |  |
| LB3 | TAGCATCTGAATTTCATAACCAATCTCGATACAC |  |
| dSpm1 | CTTATTTCAGTAAGAGTGTGGGGTTTTGG |  |
| pGC1-F | CACAAGTACTATTTTCACAC |  |
